# Isolation, Pathogenicity, and Comparative Phylogenetic Characteristics of an Intralineage Recombinant NADC34-Like PRRSV in China

**DOI:** 10.1155/2023/9929573

**Published:** 2023-09-12

**Authors:** Da-Song Xia, Tong Chang, Xin-Yi Huang, Xiao-Xiao Tian, Tao Wang, Xing-Yang Cui, Ling-Zhi Luo, Xue-Hui Cai, Yong-Bo Yang, Tong-Qing An

**Affiliations:** ^1^State Key Laboratory for Animal Disease Control and Prevention, Harbin Veterinary Research Institute, Chinese Academy of Agricultural Sciences, Harbin, China; ^2^Heilongjiang Veterinary Biopharmaceutical Engineering Technology Research Center, Harbin Veterinary Research Institute, Chinese Academy of Agricultural Sciences, Harbin, China; ^3^Heilongjiang Provincial Key Laboratory of Veterinary Immunology, Harbin Veterinary Research Institute, Chinese Academy of Agricultural Sciences, Harbin, China

## Abstract

Porcine reproductive and respiratory syndrome (PRRS), which causes reproductive failure in sows and respiratory symptoms in piglets, poses a significant threat to the global pig industry. PRRS virus (PRRSV) variants continue to emerge and spread among pigs. NADC34-like PRRSV has been imported into China in recent years and has shown potential as an endemic strain, which is of great concern. In this study, a NADC34-like PRRSV, named HLJ13 strain, was isolated from a farm where pigs experienced respiratory symptoms and abortions. Genomic analysis revealed that the HLJ13 strain was a potential recombinant of NADC34-like and NADC30-like strains, and the restriction fragment length polymorphism of HLJ13 was a novel pattern that was not yet listed. In the PRRSV HLJ13-inoculated group, the piglets showed mild clinical symptoms, such as persistent fever, and showed histopathological lesions in the lungs, and the virus was detectable at 3 and 7 days postinoculation in anal and nasal swabs, respectively. Recombination analysis revealed that interlineage recombinant events were detected in 8 out of 27 Chinese NADC34-like PRRSVs. Phylogenetic analysis showed that Chinese NADC34-like PRRSVs were distributed in two clades of lineage 1, and Chinese NADC34-like PRRSVs showed different N-glycosylation modifications in glycoproteins, especially in GP3 and GP5. These findings shed light on the genomic characteristics and pathogenicity of the NADC34-like PRRSV in China.

## 1. Introduction

Porcine reproductive and respiratory syndrome (PRRS), which induces reproductive failure in sows and respiratory symptoms in piglets, has caused high economic loss since its first report in North America in 1987. PRRS virus (PRRSV), the etiologic agent of PRRS, belongs to the member of *Arteriviridae* in the order *Nidovirales* [1]. Currently, PRRSV can be divided into two species, *Betaarterivirus suid 1* (PRRSV-1) and *Betaarterivirus suid 2* (PRRSV-2), represented by the LV and VR-2332 strains, respectively [2–4]. PRRSV is a single-stranded positive-sense RNA virus approximately 15 kb in length, with 5′-cap and 3′-polyadenylation [5]. The viral genome encodes at least 11 open reading frames (ORFs): ORF1a, ORF1b, ORF2a, ORF2b, ORFs 3–7, ORF5a, and NSP2 (TF) [6–8]. Among these, NSP2 is one of the most variable sections that can tolerate amino acid (aa) deletions or insertions [9]. For example, the PRRSV NADC34 strain has a 100 aa continuous deletion in NSP2 [10]. GP2-GP4 are the minor envelope glycoproteins of PRRSV that form heterotrimer and bind to the cellular receptor CD163 [11]. GP5, the major envelope glycoprotein, is highly variable, and its sequence is often used for genetic diversity analysis [12, 13].

PRRSV-2 can be classified into nine lineages (L1–L9) based on ORF5 [13]. Currently, PRRSV L1, L3, L5, and L8 are coprevalent in China [14]. In 2017, two NADC34-like PRRSV strains were reported in China, which belonged to L1 [15]. Epidemiological investigations have shown that NADC34-like PRRSV has become a potential endemic strain in China, which poses a high concern to the pig-production industry [16]. Clinically, the pathogenicity of piglets with different NADC34-like PRRSVs is significantly different [17–20]. However, the reason for this difference in pathogenicity remains unknown.

Recombination is one of the main approaches for the rapid evolution of RNA viruses, and different recombinant patterns are likely related to differences in viral pathogenicity [21]. Although some recombinant PRRSV strains have been reported, the recombinant characteristics of NADC34-like PRRSV are not well understood. In the present study, a recombinant NADC34-like PRRSV was isolated; then, the pathogenicity to piglets was investigated. More importantly, the phylogenetic and recombination characteristics of all available NADC34-like PRRSVs in China from 2017 to 2021 were analyzed, which contributes to the understanding of the diverse pathogenicity of different NADC34-like PRRSV strains.

## 2. Materials and Methods

### 2.1. Sample Collection and Detection

Clinical samples, including those of the lungs and lymph nodes, were collected from a farm in Heilongjiang province in 2021, where animals experienced respiratory system symptoms and abortions. The samples were analyzed by reverse transcription polymerase chain reaction (RT-PCR). Briefly, tissues were divided into pieces (approximately 0.1 g) and homogenized in 500 *μ*L phosphate-buffered saline (PBS). Then, 140 *μ*L supernatant was used for total RNA extraction and reverse-transcribed into cDNA. Specific primers were used for detection, as previously reported [22].

### 2.2. Viral Isolation and Identification

Positive tissues were used for viral isolation. The suspensions of homogenized positive tissues in PBS were filtered through 0.22 *μ*m filters and then inoculated onto porcine alveolar macrophages (PAMs). The PAMs were obtained by the lung lavage from 4-week-old specific pathogen-free (SPF) piglets and cultured in RPMI 1,640 medium (Gibco, USA) supplemented with 10% fetal bovine serum and 2% and penicillin-streptomycin at 37°C with 5% CO_2_. After 72 hr, the cultures were harvested and passaged three times. In addition, other pathogens, including porcine circovirus type 2, classical swine fever virus, and pseudorabies virus, were tested using RT-PCR or PCR. The isolated virus, named HLJ13, was identified by indirect immunofluorescence assay (IFA) using a monoclonal antibody against the M protein of PRRSV-2. The growth curve of the HLJ13 strain was measured by detecting viral copies at different times in PAMs.

### 2.3. Genomic Sequencing, Phylogenetic and Homology Analysis

Cultures of the third passage were used to sequence the genome of the HLJ13 strain. The whole-genome sequence was amplified with nine pairs of primers *(Supplementary 1)*, and the overlapping fragments were sequenced using the Sanger method, as previously described [9]. The segments were assembled using the SeqMan program in DNAStar 7.0 (DNASTAR, Madison, USA), and a full-length genome was generated. Multiple sequence alignments were performed using MAFFT [23]. The phylogenetic tree was constructed based on ORF5 of PRRSV by PhyML v3.0 [24] with 1,000 bootstrap replicates, using the general time-reversible nucleotide substitution model and a subtree pruning and rafting branch-swapping algorithm, as previously described [13]. The similarity of nucleotides and deduced amino acids between the HLJ13 strain and other representative strains was calculated using the Lasergene package (v 7.1).

### 2.4. Recombination Analysis

For recombination of interlineage, nine representative strains of L1–L9 were chosen as reference parent strains, including NADC30 (L1), XW008 (L2), MD001 (L3), EDRD-1 (L4), VR-2332 (L5), P129 (L6), SP (L7), JXA1 (L8), and MN30100 (L9), recombination events were analyzed by Simplot (v 3.5.1), RDP and phylogenetic tree, according to previous description [9]. For intralineage recombination, the full-length genome was divided into segments per 1,000 bp, which were used for BLAST in GenBank and to search for potential parent strains.

### 2.5. Pathogenicity to Piglets

Ten 4-week-old SPF piglets were obtained from the National Science and Technology Infrastructure Center (Harbin). The piglets were randomly divided into two groups: the inoculated group (*n* = 5) and the control group (*n* = 5). The infected group was inoculated intramuscularly (1 mL) and intranasally (2 mL) with the HLJ13 strain, while the control group was inoculated with Dulbecco's modified eagle medium at the same volume and via the same route. The titer of HLJ13 was 3 × 10^5^ TCID_50_/mL. After the viral challenge, clinical symptoms and rectal temperature were recorded daily. The clinical symptom scores were calculated weekly according to previously published criteria [25]. Blood, nasal, and anal swabs were collected at 0, 3, 7, 10, 14, and 21 days postinoculation (dpi), and all surviving piglets were euthanized at 21 dpi. Piglets were weighed at 0 and 21 dpi to calculate the average weight gain rate. In addition, thymuses were weighed to calculate the thymus/weight ratio (g/kg). Tissue samples, such as heart, liver, spleen, lung, kidney, thymus, tonsil, and lymph nodes, were collected. Lung samples from each pig were fixed in 4% paraformaldehyde for hematoxylin and eosin staining.

### 2.6. Viremia and Viral Distribution Test by Real-Time Quantitative PCR (RT-qPCR)

The level of viremia and viral distribution in the tissues were determined using RT-qPCR. The collected tissues were processed as previously described [17]. The samples (0.1 g) were homogenized with 500 *μ*L of PBS, and then 140 *μ*L supernatant was used for RNA extraction according to the manufacturer's instructions (TIANGEN, Beijing, China). A serum sample (140 *μ*L) was also acquired for RNA extraction. RT-qPCR was performed, as previously described [26].

### 2.7. Viral Shedding

The nasal and anal swabs collected on different days postinoculation were homogenized with 500 *μ*L PBS, then 140 *μ*L supernatant was used for RNA extraction. The viral load of the swabs was also detected by RT-qPCR to evaluate the level of viral shedding.

### 2.8. Detection of PRRSV Antibodies

Serum samples collected at 0, 7, 10, 14, and 21 dpi were used to detect N protein antibodies of PRRSV. The analysis was performed according to the manufacturer's instructions using a HerdCheck PRRS × 3 ELISA kit (IDEXX, USA). *S*/*P* > 0.4 was considered as the threshold of serological positivity.

### 2.9. Datasets and Restriction Fragment Length Polymorphism (RFLP) Types of NADC34-Like Strains in China

NADC34-like strains reported in China in 2017–2021, which have a continuous 100-aa deletion in NSP2 similar to the IA/2014/NADC34 strain, were collected from GenBank. The provinces and clinical symptoms of these strains are listed (Table 1). The RFLP patterns based on ORF5 were also analyzed according to previously published criteria [27]. According to the positions of the restriction enzymes *Mlu* Ⅰ, *Hinc* Ⅱ, and *Sac* Ⅱ in ORF5, PRRSV can be divided into different types. If the restriction enzyme positions were not in the criteria, “?” was used to show the pattern at this position of the restriction enzyme.

### 2.10. Glycosylation Site Prediction

The N-glycosylation sites in the glycoprotein were predicted using the website https://services.healthtech.dtu.dk/service.php?NetNGlyc-1.0. In detail, the default threshold of 0.5 was used as the cutoff of potential N-glycosylation sites. The threshold of 0.75 served as the criterion for high confidence of the N-glycosylation. The level of potential N-glycosylation in glycoproteins was calculated using GraphPad Prism (v 8.0) and was depicted using a heatmap.

### 2.11. Statistical Analysis

Data were statistically analyzed using a two-way analysis of variance test in GraphPad Prism, and *p* < 0.05 was considered as statistically significance and is indicated as ns, *p* > 0.05;  ^*∗*^, *p* < 0.05;  ^*∗∗*^, *p* < 0.01;  ^*∗∗∗*^, *p* < 0.001;  ^*∗∗∗∗*^, *p* < 0.0001.

## 3. Results

### 3.1. Viral Isolation and Identification

The sample was determined to be positive for PRRSV through RT-PCR analysis of ORF5. The filtrate of the positive sample homogenate was subsequently incubated with PAMs for three passages. The isolated PRRSV strain, designated HLJ13, was confirmed using RT-PCR *(Supplementary 2)*. Interestingly, we found that the HLJ13 strain could replicate in PAMs *(Supplementary 2)* but not in Marc-145 cells (data not shown). The multistep growth curve revealed that the viral copies increased gradually after infection and reached 9.4 × 10^4^ copies/*μ*L at 96 hr postinfection. Furthermore, IFA was performed to verify the presence of the HLJ13 strain in infected cells, and specific fluorescence was observed in HLJ13 strain-infected PAMs but not in the control group (*Supplementary 2*).

### 3.2. Phylogenetic Analysis and Genome Characteristic of PRRSV HLJ13 Strain

**Figure 1:**
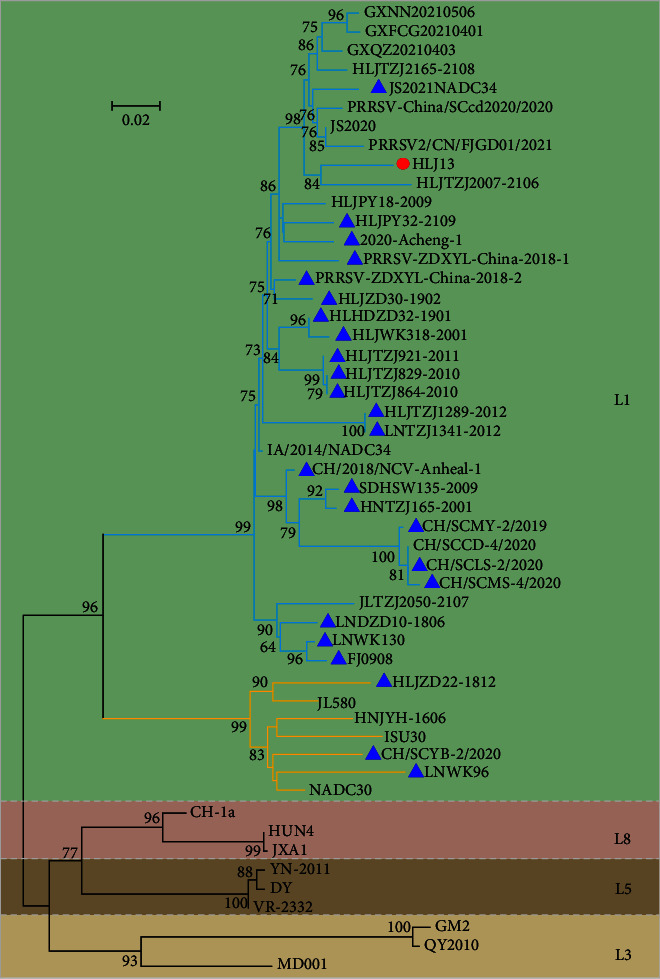
Phylogenetic analysis of HLJ13 and other NADC34-like strains isolated in China. The phylogenetic tree was constructed based on ORF5. The red circle represents the HLJ13 strain, while the blue triangle shows other NADC34-like strains in China collected in GenBank. In the L1 phylogenetic tree, the branches of sublineage 1.5 are depicted in blue and sublineage 1.8 in yellow.

Phylogenetic analysis based on the ORF5 gene revealed that the HLJ13 strain was located in sublineage 1.5, which also contained the IA/2014/NADC34 strain (Figure 1). The NSP2 of the HLJ13 strain contains a continuous 100 aa deletion at position 328–427, which is the same feature as that of the IA/2014/NADC34 strain (*Supplementary 3*). Multiple sequence alignment analysis showed that the HLJ13 strain shared high nucleotide identity with the IA/2014/NADC34 strain (93.7%) compared to other strains *(Supplementary 4)*. In particular, based on the NSP2 gene, which is one of the most variable proteins in PRRSV, the HLJ13 strain shared 94.2% homology with the IA/2014/NADC34 strain. In addition, a comparison of structural proteins showed that the HLJ13 strain had a higher amino acid identity with the IA/2014/NADC34 strain *(Supplementary 5)*. However, RFLP-analysis based on the ORF5 gene revealed the *Mlu* Ⅰ, *Hinc* Ⅱ, *Sac* Ⅱ restriction enzyme positions of the HLJ13 strain (*Mlu* Ⅰ, *Hinc* Ⅱ, *Sac* Ⅱ = NA; 88, 219, 596; 24, 555) that have not been reported previously. Thus, the RFLP type of the HLJ13 strain was named 1–?– 4 because of the *Hinc* Ⅱ enzyme position being out of the criteria, which is different from the IA/2014/NADC34 strain (1–7–4).

### 3.3. Recombination Characteristic of HLJ13 Strain

**Figure 2:**
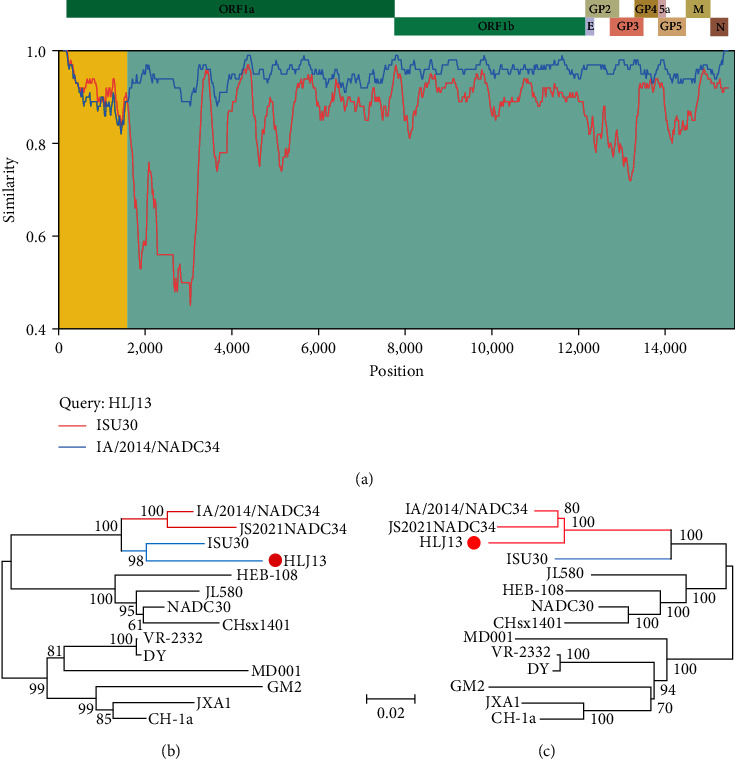
Recombination analysis of HLJ13 strain. (a) The similarity plots were generated by Simplot. The recombination breakpoint was determined in nt 1,561 (referenced to VR-2332 genome). Phylogenetic trees were constructed based on the two parts of 1–1,561 (b) and 1,562–15,467 (c), which were divided by the breakpoint.

To investigate potential recombination events, recombination analysis was performed using the SimPlot and RDP software. Based on the similarity plot, the recombination breakpoint (nucleotides (nt) 1,561) was located in NSP2 of the HLJ13 strain (Figure 2), which divided its genome into two regions: region A (nt 1–1,561) and region B (nt 1,562–15,467). Phylogenetic trees were constructed based on these two regions, and the results showed that region A was closely related to the ISU30 strain (a NADC30-like PRRSV, sublineage 1.8), whereas region B was closely related to the IA/2014/NADC34 strain (sublineage 1.5). Hence, the aforementioned findings suggest that there was an intralineage recombination event in the genome of the HLJ13 strain, which took the NADC34-like strain as the backbone and recombined with the NADC30-like strain.

### 3.4. Clinical Symptom and Pathological Lesion of HLJ13-Inoculated Piglets

**Figure 3:**
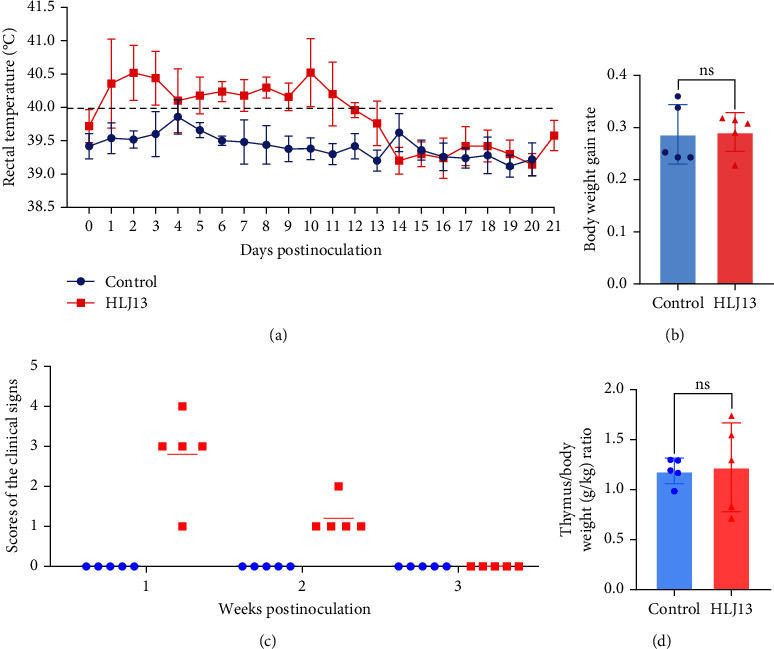
Clinical symptoms of piglets in inoculated and control groups. (a) Rectal temperature. The daily rectal temperature was measured, and ≥40.0°C was defined as a fever. (b) Body weight gain rate. The body weight of the HLJ13-inoculated and control group was recorded at 0 and 21 dpi. ns, no significant difference. (c) Scores of clinical symptoms. (d) The ratio of thymus/body weight (g/kg) ratio. At 21 dpi, the weight of the thymus and body was determined, and the ratio of thymus/body weight (g/kg) was measured (ns, *p* > 0.05;  ^*∗*^, *p* < 0.05;  ^*∗∗*^, *p* < 0.01;  ^*∗∗∗*^, *p* < 0.001;  ^*∗∗∗∗*^, *p* < 0.0001).

**Figure 4:**
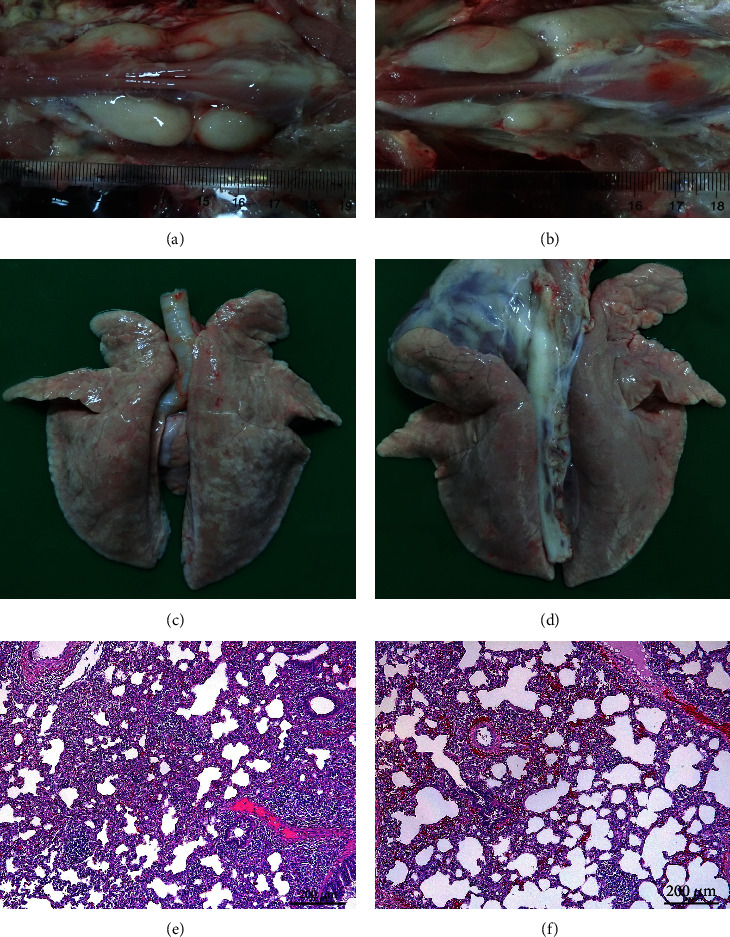
Pathological lesion of thymus and lung. (a), (c), and (e) show the thymus, lung, and histological assessment of the lung in the HLJ13-inoculated group, respectively. (b), (d), and (f) Indicate thymus, lungs, and histological lesions of the lung from the control group, respectively.

Clinical signs of the piglets challenged with the HLJ13 strain included a persistent fever (>40°C) from 1 to 12 days postinoculation (dpi), with a peak of 40.5°C at 2 dpi, but without evidence of reduced bodyweight and death (Figures 3(a) and 3(b)). Moreover, several piglets showed inappetence and lethargy in the first week after inoculation, but these clinical symptoms resolved quickly (Figure 3(c)). Furthermore, no significant change in the thymus/body weight ratio (Figure 3(d)) or thymus atrophy was observed in the HLJ13 strain-infected group compared to the control group (Figures 4(a) and 4(b)). Lung tissues of the HLJ13-inoculated group showed mild macroscopic lesions, such as pulmonary emphysema (Figure 4(c)), while no visible lesions were observed in the control group (Figure 4(d)). Histopathological examination of the lungs revealed infiltration of numerous inflammatory cells and proliferation of alveolar epithelia, leading to widened alveolar septa in HLJ13-infected piglets (Figure 4(e)). In contrast, the lungs in the uninfected control group showed no macroscopic lesions or inflamed cells (Figure 4(f)).

### 3.5. Detection of PRRSV Antibodies

**Figure 5:**
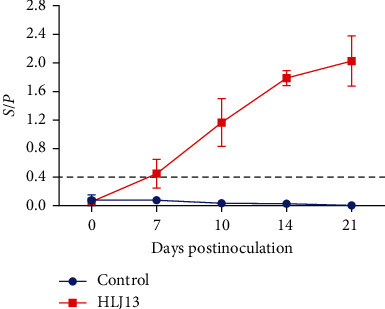
Detection of PRRSV antibody. Serum was used to detect PRRSV N protein antibodies using the IDEXX HerdCheck ELISA kit and *S*/*P* > 0.4 as the threshold of seroconversion to positive.

Piglet serum samples were collected for PRRSV N protein antibody measurements. In the HLJ13-inoculated group, 3/5 piglets became seroconverted (*S*/*P* > 0.4) at 7 dpi and all piglets were seropositive at 10 dpi. Subsequently, the levels of PRRSV antibodies gradually increased. In particular, the mean *S*/*P* value reached about 2.0 at 21 dpi, whereas the control group was consistently negative (*S*/*P* < 0.4) (Figure 5).

### 3.6. Viremia and Viral Distribution in Different Tissues

**Figure 6:**
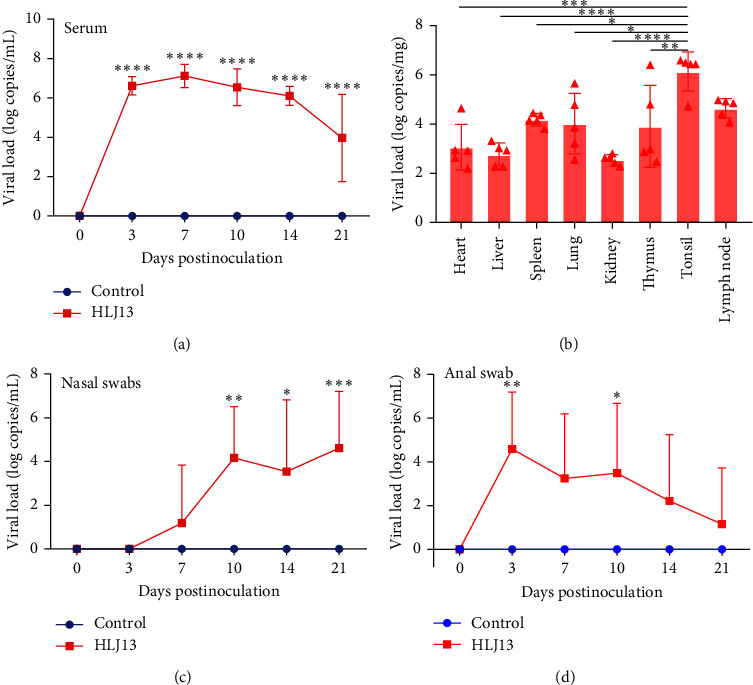
Viremia, viral tissue distribution, and shedding. Viremia in serum (a) and viral distribution in tissues (b) of piglets were detected. (c) (d) Show the virus shedding in nasal swabs and anal swabs, respectively. The virus in the HLJ13-inoculated and control groups was detected by RT-qPCR (ns, *p* > 0.05;  ^*∗*^, *p* < 0.05;  ^*∗∗*^, *p* < 0.01;  ^*∗∗∗*^, *p* < 0.001;  ^*∗∗∗∗*^, *p* < 0.0001).

To examine the viral replication ability of the HLJ13 strain in vivo, the levels of viremia and viral tissue distribution in piglets were assessed. The analysis of viremia showed that plasma viral levels increased rapidly postinoculation and reached a peak at 7 dpi (2.34 × 10^7^copies/mL), followed by a gradual decrease (Figure 6(a)). In addition, the measurement of viral load in tissue samples showed that the HLJ13 strain was widely distributed in all collected tissues, particularly in the tonsils (2.52 × 10^6^ copies/mg) and lymph nodes (5.72 × 10^5^ copies/mg) (Figure 6(b)).

### 3.7. Viral Shedding in Respiratory and Digestive Tract

Viral shedding in the respiratory and digestive tracts of the HLJ13-inoculated group was markedly different. In the nasal swabs, viral RNA was detectable at 7 dpi, and the level of viral load increased gradually and reached a peak at 21 dpi (5.96 × 10^5^ copies/mL) (Figure 6(c)). In contrast, the highest viral load was detected at 3 dpi in anal swabs (6.56 × 10^5^ copies/mL), which was earlier than that in nasal swabs (Figure 6(d)). Samples in the control group remained negative throughout the experimental period.

### 3.8. Comparative Phylogenetic Characteristics of Chinese NADC34-Like PRRSV in 2017–2021

To explore the genetic diversity, a comparative phylogeny, RFLP pattern, deletion polymorphisms of the NSP2 protein, and recombination analysis of Chinese NADC34-like PRRSV strains from 2017 to 2021 were performed. Phylogenetic analysis based on the ORF5 gene revealed that most NADC34-like PRRSVs were classified into sublineage 1.5 with the IA/2014/NADC34 strain, whereas three NADC34-like strains were located in sublineage 1.8 along with the representative NADC30 strain (Figure 1(a)). These data suggest that the genetic diversity of NADC34-like PRRSV is closely related to viral recombination.

**Figure 7:**
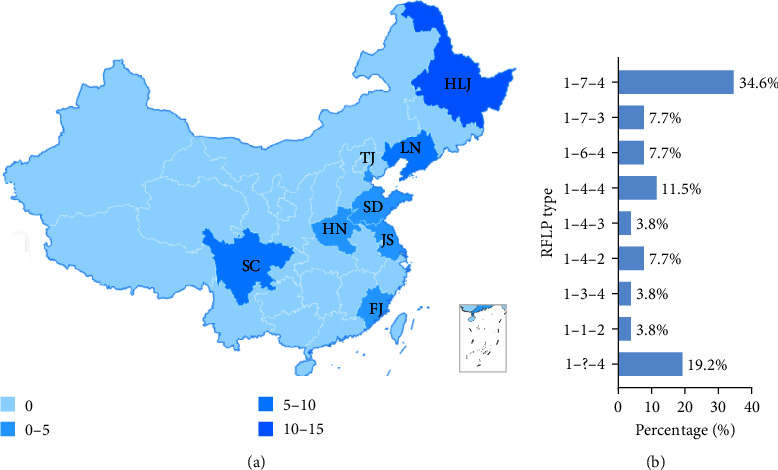
Geographical distribution and RFLP analysis of NADC34-like PRRSVs in China. (a) Geographical distribution. The number of NADC34-like PRRSV in different provinces was counted and illustrated by the shade of color. HLJ, Heilongjiang *n* = 14, LN, Liaoning *n* = 4, TJ, Tianjin *n* = 1, SD, Shandong *n* = 1, HN, Henan *n* = 1, JS, Jiangsu *n* = 1, SC, Sichuan *n* = 4, FJ, Fujian *n* = 1. (b) RFLP analysis based on ORF5 of NADC34-like PRRSVs.

Geographical distribution analysis revealed that NADC34-like strains were detected mainly in the major pig-production provinces of China, especially in Heilongjiang (*n* = 14) and Liaoning (*n* = 4) (Figure 7(a)). RFLP patterns were also identified based on the ORF5 gene of the NADC34-like PRRSV. The results showed that the most common patterns were 1–7–4 (34.6%), followed by 1–?–4 (19.2%) and 1–4–4 (11.5%), implying RFLP-type diversity of NADC34-like PRRSV (Figure 7(b)).

Amino acid sequence alignments revealed that all NADC34-like PRRSV showed a 100 aa continuous deletion in 328–427 aa of NSP2. Moreover, some NADC34-like strains had irregular deletions in other regions of NSP2. For example, the NSP2 of PRRSV 2020-Acheng-1 strain had an additional 49 aa continuous deletion at 469-517 aa, while the CH/SCMY-2/2019 strain and CH/2018/NCV-Anheal-1 strain had one aa deletion at aa 484 and 497 of NSP2, respectively. The HLJWK318-2001 strain had two aa deletions in 152–153 aa; the HNTZJ165-2001 and HLJPY 32-2109 strains had four and eight aa deletions in 484–487 aa and 499–506 aa of NSP2, respectively (*Supplementary 3*).

Notably, a small number (8/27) of the Chinese NADC34-like strains were found to have undergone interlineage recombination events (Table 2; *Supplementary 6*). The HLJ13, LNWK96, LNWK130, and LNDZD10-1806 strains showed similar recombination patterns, which were based on the IA/2014/NADC34 strain as the major parent strain, with a minor parent from the ISU30-like strain. Other recombinants included the CH/SCYB-2/2020, CH/SCMY-2/2020, and HLJZD22-1812 strains, with the IA/2014/NADC34 strain as the major parent and the CHsx1401, FJWQ16, and JL580 strains as the minor parent, respectively. Thus, recombination patterns have become more complex since the first NADC34-like strain was reported in China, suggesting that NADC34-like PRRSV can increase genetic diversity through recombination with local strains (Table 2).

### 3.9. GP5 Amino Acid Variation of NADC34-Like PRRSV

**Figure 8:**
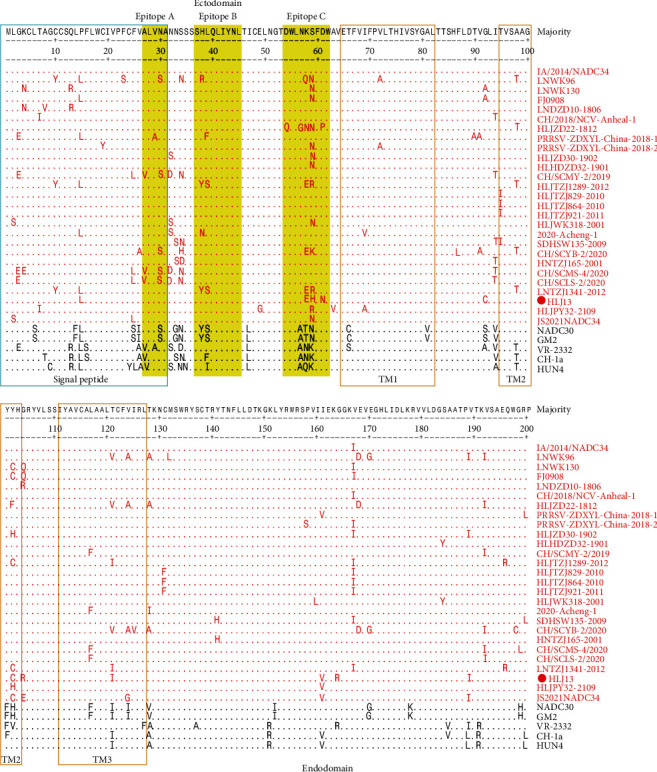
Alignment of GP5 of NADC34-like PRRSV in China. Epitopes A, B, and C were indicated by shades of yellow. In the alignment, the residues and names of Chinese NADC34-like strains were marked with red.

The envelope glycoprotein GP5 has been proposed to be one of the most variable regions of the PRRSV genome and plays an important role in the production of neutralizing antibodies. To further investigate the genetic diversity of NADC34-like PRRSV, an alignment analysis of GP5 amino acid sequences between Chinese NADC34-like isolates and other lineages of PRRSV strains was performed. Several amino acid mutations were observed in the three linear epitopes, especially in epitope C. Moreover, two amino acid positions (47 and 57) were found to be conserved in GP5 of Chinese NADC34-like isolates compared with other lineages of PRRSV strains. Furthermore, we found that most amino acids were conserved between the HLJ13 and IA/2014/NADC34 strains except nine amino acid mutations. Interestingly, three amino acid substitutions in GP5 of the HLJ13 strain were located in epitope C (58, 59, and 61) (Figure 8).

### 3.10. N-Glycosylation Site in Glycoproteins of NADC34-Like PRRSV

**Figure 9:**
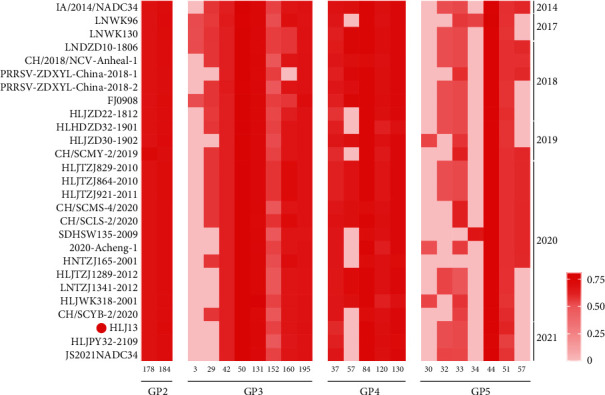
N-glycosylation site prediction in PRRSV glycoprotein. The color indicates the probability of N-glycosylation in different positions. When the probability of prediction is >0.5, there is a potential N-glycan at that site.

Glycoprotein glycosylation is crucial for viral invasion and immune evasion. In GP2, the N-glycosylation patterns at sites N178 and N184 were highly conserved among NADC34-like PRRSVs. For GP3, N50, N131, and N195 were the most conserved N-glycosylation sites. In contrast, the percentage in N3 and N29 of NADC34-like strains decreased in recent years. Only the PRRSV-ZDXYL-China-2018-1 strain lost glycans in N160 of GP3. As for GP4, N57 may not be necessary, and the other four sites were conserved in these isolates. Moreover, in GP5, the most conserved N-glycosylation site was observed at N44, which is located in epitope B. Only LNWK96, SDHSW135-2009 strain and 2020-Acheng-1, HLJWK318-2001, HLJZD30-1902 strains were modified by glycosylation at N34 and N30, respectively. In addition, the HLJ13 strain showed the same N-glycosylation pattern as most Chinese NADC34-like strains, but three N-glycans were lost in GP3 (N29), GP4 (N57), and GP5 (N57) compared with IA/2014/NADC34 (Figure 9).

## 4. Discussion

In 2014, the PRRSV IA/2014/NADC34 strain, with a unique continuous 100 aa deletion in NSP2, was first isolated in the United States. The strain showed high pathogenicity in 3-week-old piglets, causing severe clinical signs such as high levels of pyrexia and significant weight gain [10]. In 2017, two NADC34-like strains were first isolated from Liaoning province in China, LNWK96 and LNWK130, showing the same NSP2 deletion pattern as the IA/2014/NADC34 strain [15]. Since then, an increasing number of NADC34-like strains have been reported, implying a pandemic potential in China [16, 28, 29]. However, studies on the genetic diversity and pathogenicity of NADC34-like PRRSV in China are still inadequate.

In this study, we isolated a recombinant NADC34-like PRRSV HLJ13 strain. The viral distribution results revealed the highest viral load in the tonsils, and previous reports indicated that the viral level could be detected in the tonsils despite no detection in serum; thus, the tonsils were a long-term carrier of PRRSV [30, 31]. Moreover, the nasal viral shedding level in the HLJ13-inoculated group persistently increased until 21 dpi. Persistent viral shedding caused by HLJ13 may be a topic of concern. In 2020, a highly pathogenic PRRSV, RFLP 1–4–4 L1C variant, emerged in the midwest region of the United States. Subsequent studies revealed that the RFLP 1–4–4 L1C variant was a recombinant of NADC34 and other PRRSV strains [32, 33]. Thus, the pathogenicity of NADC34-like PRRSV may be enhanced via recombination. Currently, the epidemic caused by Chinese NADC34-like strains is still in its early stages and shows simple genetic diversity. Therefore, it is important to monitor the prevalence and recombination of NADC34-like PRRSVs in China. In 2022, a nonrecombinant NADC34-like PRRSV JS2021 strain was identified as highly pathogenic, with a high mortality rate of 75%. Thus, although the NADC34-like PRRSV in China shows high homology, its pathogenicity is diverse, which may be due to mutations or recombination.

Glycosylation is important for viral biological processes, including viral protein folding conformation, entry into host cells, and immune evasion [34]. A previous study reported that GP3 is a heavily glycosylated protein of PRRSV; N42, N50, and N131 of GP3 are necessary for viral infectivity, while deletion of the N131 glycosylation site resulted in high susceptibility to neutralization of the strain [35]. Mutations at any two of the four predicted N-glycosylation sites (N37, N84, N120, and N130) in GP4 had a significant impact on infectious PRRSV recovery [36]. Glycosylation in GP5 may be responsible for the evasion of antibody neutralization due to glycan shielding [37]. In this study, N44 and N51 of GP5 were conserved in all analyzed NADC34-like PRRSVs, whereas N30 was variable, and there was no glycosylation at N35. The HLJ13 strain lost the N-glycan at residue N57, which is located in epitope C, compared to the IA/2014/NADC34 strain. Epitope C acted as the target for homologous neutralization; mutations in epitope C led to immune evasion from homologous neutralization antibodies [38].

In summary, a recombinant NADC34-like PRRSV HLJ13 strain with a novel RFLP pattern was isolated, and its pathogenicity in piglets was examined. Furthermore, recombination, RFLP patterns, and all available NADC34-like PRRSVs in China were analyzed, and the results revealed complex genetic diversity among NADC34-like PRRSV strains. This result is helpful for understanding the pathogenicity and genetic diversity of NADC34-like PRRSV.

## Figures and Tables

**Table 1:** Information of NADC34-like strains in China.

No.	Strains	Accession no.	Isolated year	Province	Clinical signs	PMID
1	LNWK96	MG860516	2017	Liaoning	Approximate abortion rates of 20%, mortality rates of 10%	30080663
2	LNWK130	MG913987	2017	Liaoning	Approximate abortion rates of 30%, mortality rates of 10%	30080663
3	FJ0908	MK202794	2018	Fujian	High abortion rate (25%) and mortality (40%)	31637126
4	LNDZD10-1806	MN648054	2018	Liaoning	Abortion rates of 10%	32037673
5	CH/2018/NCV-Anheal-1	MH370474	2018	Heilongjiang	—	—
6	HLJZD22-1812	MN648450	2018	Heilongjiang	Abortion rates of 20%	32037673
7	PRRSV-ZDXYL-China-2018-1	MK453049	2018	Heilongjiang	Pregnant sows had abortions and stillbirths, and the piglets showed high fever and obvious respiratory signs, with an approximately 80% mortality	31862391
8	PRRSV-ZDXYL-China-2018-2	MK453050	2018	Heilongjiang		
9	HLHDZD32-1901	MN648449	2019	Heilongjiang	Abortion rates of 10%	32037673
10	HLJZD30-1902	MN648055	2019	Heilongjiang	Abortion rates of 10%	32037673
11	CH/SCMY-2/2019	OL771205	2019	Sichuan	Abortion rates of 10%	35090082
12	HLJTZJ1289-2012	OL516352	2020	Heilongjiang	—	35182461
13	LNTZJ1341-2012	OL516360	2020	Liaoning	—	35182461
14	HLJTZJ829-2010	OL516349	2020	Heilongjiang	—	35182461
15	HLJTZJ864-2010	OL516350	2020	Heilongjiang	—	35182461
16	HLJTZJ921-2011	OL516351	2020	Heilongjiang	—	35182461
17	HLJWK318-2001	OL516357	2020	Heilongjiang	—	35182461
18	2020-Acheng-1	MW079495	2020	Heilongjiang	—	35998397
19	SDHSW135-2009	OL516361	2020	Shandong	—	35182461
20	CH/SCYB-2/2020	OL771209	2020	Sichuan	—	35090082
21	HNTZJ165-2001	OL516358	2020	Henan	—	35182461
22	CH/SCMS-4/2020	OL771208	2020	Sichuan	Abortion rates of 10%	35090082
23	CH/SCLS-2/2020	OL771207	2020	Sichuan	Abortion rates of 10%	35090082
24	HLJ13		2021	Heilongjiang	—	This study
25	HLJPY32-2109	OL516348	2021	Heilongjiang	—	35182461
26	JS2021NADC34	MZ820388	2021	Jiangsu	—	34786872
27	TJnh2021	—	2021	Tianjin	75% morbidity and 40% mortality	35119777

**Table 2:** Recombination analysis of Chinese NADC34-like PRRSV strains.

Strains name	Recombinant breakpoint	Potential major parent	Potential minor parent
LNWK96	1–14,41(5′UTR-NSP2), 13,281–15,529 (ORF4-3′UTR)	IA/2014/NADC34	ISU30, NADC30
LNWK130	1–1,480 (5′UTR-NSP2)	IA/2014/NADC34	ISU30
HLJZD22-1812	14,021–15,481 (ORF5-3′UTR)	IA/2014/NADC34	JL580
LNDZD10-1806	1–1,441 (5′UTR-NSP2)	IA/2014/NADC34	ISU30
CH/SCMY-2/2019	13,381–13,861 (ORF4) 14,461–15,447 (ORF5-3′UTR)	IA/2014/NADC34	FJWQ16
CH/SCYB-2/2020	13,881–14,521 (ORF4-6)	IA/2014/NADC34	CHsx1401
TJnh2021	12,196–13,628 (ORF2-5)	IA/2014/NADC34	QYYZ
HLJ13	1–1,561 (5′UTR-NSP2)	IA/2014/NADC34	ISU30

## Data Availability

The data and material that support the findings of this study can be provided through the corresponding author upon reasonable request.

## Supplementary Materials

Supplementary 1 Nine pairs of primers for amplify the whole genome of HLJ13.

Supplementary 2 Identification and isolation of HLJ13 strain.

Supplementary 3 Amino acid alignment of NSP2. The 100 aa continuous deletion of NSP2 was highlighted by yellow box, while the additional deletion was represented by blue box. The names of PRRSV strains were revealed in the righted, and the isolated HLJ13 strain in this study was marked by red circle. The labeled amino acid positions were referenced to VR-2332.

Supplementary 4 The nucleotide homology of HLJ13 represents PRRSV strains.

Supplementary 5 The homology of amino acid between HLJ13 and represent strains in different lineage and sublineages.

Supplementary 6 Recombination analysis of NADC34-like strains in China. The preformation was the same as the HLJ13 strain. The left and right sequence of breakpoints were alignment and phylogenetic analysis. The TJnh2021 strain refers to this paper (PMID: 35119777).
