# Supplementary material for: Isolation, Pathogenicity, and Comparative Phylogenetic Characteristics of an Intralineage Recombinant NADC34-Like PRRSV in China
Source: Transbound Emerg Dis. 2023 Sep 12;2023:9929573. doi: 10.1155/2023/9929573 (PMC12017108; doi:10.1155/2023/9929573)
Supplement: Supplementary 1 — Nine pairs of primers for amplify the whole genome of HLJ13. [file 9929573.f1.docx]

**Isolation, pathogenicity and comparative phylogenetic characteristics of an intra-lineage recombinant NADC34-like PRRSV in China**

Da-Song Xia^1, #^, Tong Chang^1, #^, Xin-Yi Huang^1^, Xiao-Xiao Tian^1^, Tao Wang^1^, Xing-Yang Cui^1^, Ling-Zhi Luo^1^, Xue-Hui Cai^1,3^, Yong-Bo Yang^1,3, *^, Tong-Qing An^1,2, *^

^1^ State Key Laboratory for Animal Disease Control and Prevention, Harbin Veterinary Research Institute, Chinese Academy of Agricultural Sciences, Harbin, China.

^2^ Heilongjiang Provincial Key Laboratory of Veterinary Immunology, Harbin Veterinary Research Institute, Chinese Academy of Agricultural Sciences, Harbin, China.

^3^ Heilongjiang Veterinary Biopharmaceutical Engineering Technology Research Center, Harbin Veterinary Research Institute, Chinese Academy of Agricultural Sciences, Harbin, China.

* Corresponding author: Dr. Tong-Qing An

State Key Laboratory for Animal Disease Control and Prevention

Harbin Veterinary Research Institute, Chinese Academy of Agricultural Sciences

No. 678 Haping Road, Xiangfang District, Harbin, 150069, China

Tel.: +86-451-51051765; Fax: +86-451-51997166.

E-mail: [antongqing@caas.cn](mailto:antongqing@caas.cn)

Dr. Yong-Bo Yang

State Key Laboratory for Animal Disease Control and Prevention

Harbin Veterinary Research Institute, Chinese Academy of Agricultural Sciences

No. 678 Haping Road, Xiangfang District, Harbin, 150069, China

Tel.: +86-451-51051762; Fax: +86-451-51997166.

E-mail: [yangyongbo@caas.cn](mailto:yangyongbo@caas.cn)

Table S3: nine pairs of primers for amplify the whole genome of HLJ13

| Primer name | Primer sequence (5′-3′) | Position in genome (bp) | Product size (bp) |
| --- | --- | --- | --- |
| HLJ13-A-F | GCACTGCTTTACGGTCTCTCC | 1-1556 | 1556 |
| HLJ13-A-R | GTCCAGTGCTCGCCTTC |  |  |
| HLJ13-B-F | GTCAGTGCTAACAAGGC | 1284-3147 | 1864 |
| HLJ13-B-R | AACATCCTCGGTGGCAA |  |  |
| HLJ13-C-F | ATGCTAACCTGGCGCAAC | 2952-4514 | 1562 |
| HLJ13-C-R | GCCATCAAGCAAGCAAC |  |  |
| HLJ13-D-F | CCGAGAAACCTATTGCG | 4111-5895 | 1784 |
| HLJ13-D-R | GACAGCTGGGAGTATCTC |  |  |
| HLJ13-E-F | GAATTCTTCGCTGGACCCA | 5649-7344 | 1695 |
| HLJ13-E-R | GCCCCAGGGTAAAGGT |  |  |
| HLJ13-F-F | CCGGTAGGAAGTTTCTTG | 7051-9231 | 2180 |
| HLJ13-F-R | CGTCAAGACCACAGGCAG |  |  |
| HLJ13-G-F | GGCTGCAATACTCATGGAC | 8998-11513 | 2515 |
| HLJ13-G-R | AAACGCTTCATTGTAATCCTC |  |  |
| HLJ13-H-F | GGTGAGGACTGGGAGGATT | 11480-13677 | 2189 |
| HLJ13-H-R | CGCAGACGGCGTAAATG |  |  |
| HLJ13-I-F | TTGCCTTTTTTGTGGTGTATC | 13370-14955 | 1586 |
| HLJ13-I-R | AATTTCGGCCGCATGGTTCTC |  |  |
