# Supplementary material for: Isolation, Pathogenicity, and Comparative Phylogenetic Characteristics of an Intralineage Recombinant NADC34-Like PRRSV in China
Source: Transbound Emerg Dis. 2023 Sep 12;2023:9929573. doi: 10.1155/2023/9929573 (PMC12017108; doi:10.1155/2023/9929573)
Supplement: Supplementary 3 — Amino acid alignment of NSP2. The 100 aa continuous deletion of NSP2 was highlighted by yellow box, while the additional deletion was represented by blue box. The names of PRRSV strains were revealed in the righted, and the isolated HLJ13 strain in this study was marked by red circle. The labeled amino acid positions were referenced to VR-2332. [file 9929573.f3.docx]

**Isolation, pathogenicity and comparative phylogenetic characteristics of an intra-lineage recombinant NADC34-like PRRSV in China**

Da-Song Xia^1, #^, Tong Chang^1, #^, Xin-Yi Huang^1^, Xiao-Xiao Tian^1^, Tao Wang^1^, Xing-Yang Cui^1^, Ling-Zhi Luo^1^, Xue-Hui Cai^1,3^, Yong-Bo Yang^1,3, *^, Tong-Qing An^1,2, *^

^1^ State Key Laboratory for Animal Disease Control and Prevention, Harbin Veterinary Research Institute, Chinese Academy of Agricultural Sciences, Harbin, China.

^2^ Heilongjiang Provincial Key Laboratory of Veterinary Immunology, Harbin Veterinary Research Institute, Chinese Academy of Agricultural Sciences, Harbin, China.

^3^ Heilongjiang Veterinary Biopharmaceutical Engineering Technology Research Center, Harbin Veterinary Research Institute, Chinese Academy of Agricultural Sciences, Harbin, China.

* Corresponding author: Dr. Tong-Qing An

State Key Laboratory for Animal Disease Control and Prevention

Harbin Veterinary Research Institute, Chinese Academy of Agricultural Sciences

No. 678 Haping Road, Xiangfang District, Harbin, 150069, China

Tel.: +86-451-51051765; Fax: +86-451-51997166.

E-mail: [antongqing@caas.cn](mailto:antongqing@caas.cn)

Dr. Yong-Bo Yang

State Key Laboratory for Animal Disease Control and Prevention

Harbin Veterinary Research Institute, Chinese Academy of Agricultural Sciences

No. 678 Haping Road, Xiangfang District, Harbin, 150069, China

Tel.: +86-451-51051762; Fax: +86-451-51997166.

E-mail: [yangyongbo@caas.cn](mailto:yangyongbo@caas.cn)

**Fig S2**


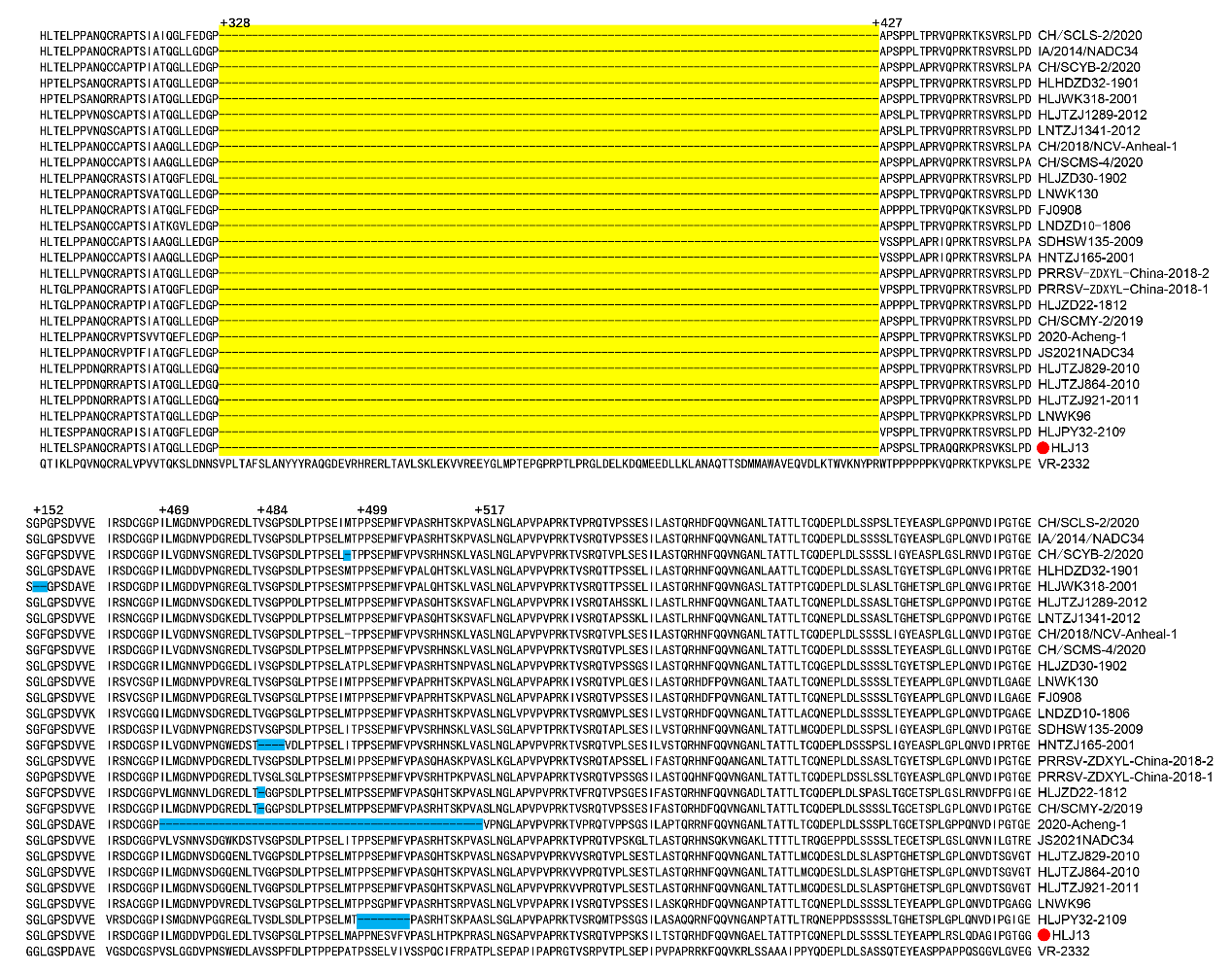


Fig S2: Amino acid alignment of NSP2. The 100 aa continuous deletion of NSP2 was highlighted by yellow box, while the additional deletion was represented by blue box. The names of PRRSV strains were revealed in the righted and the isolated HLJ13 strain in this study was marked by red circle. The labeled amino acid positions were reference to VR-2332.
