# Supplementary material for: Isolation, Pathogenicity, and Comparative Phylogenetic Characteristics of an Intralineage Recombinant NADC34-Like PRRSV in China
Source: Transbound Emerg Dis. 2023 Sep 12;2023:9929573. doi: 10.1155/2023/9929573 (PMC12017108; doi:10.1155/2023/9929573)
Supplement: Supplementary 4 — The nucleotide homology of HLJ13 represents PRRSV strains. [file 9929573.f4.docx]

**Isolation, pathogenicity and comparative phylogenetic characteristics of an intra-lineage recombinant NADC34-like PRRSV in China**

Da-Song Xia^1, #^, Tong Chang^1, #^, Xin-Yi Huang^1^, Xiao-Xiao Tian^1^, Tao Wang^1^, Xing-Yang Cui^1^, Ling-Zhi Luo^1^, Xue-Hui Cai^1,3^, Yong-Bo Yang^1,3, *^, Tong-Qing An^1,2, *^

^1^ State Key Laboratory for Animal Disease Control and Prevention, Harbin Veterinary Research Institute, Chinese Academy of Agricultural Sciences, Harbin, China.

^2^ Heilongjiang Provincial Key Laboratory of Veterinary Immunology, Harbin Veterinary Research Institute, Chinese Academy of Agricultural Sciences, Harbin, China.

^3^ Heilongjiang Veterinary Biopharmaceutical Engineering Technology Research Center, Harbin Veterinary Research Institute, Chinese Academy of Agricultural Sciences, Harbin, China.

* Corresponding author: Dr. Tong-Qing An

State Key Laboratory for Animal Disease Control and Prevention

Harbin Veterinary Research Institute, Chinese Academy of Agricultural Sciences

No. 678 Haping Road, Xiangfang District, Harbin, 150069, China

Tel.: +86-451-51051765; Fax: +86-451-51997166.

E-mail: [antongqing@caas.cn](mailto:antongqing@caas.cn)

Dr. Yong-Bo Yang

State Key Laboratory for Animal Disease Control and Prevention

Harbin Veterinary Research Institute, Chinese Academy of Agricultural Sciences

No. 678 Haping Road, Xiangfang District, Harbin, 150069, China

Tel.: +86-451-51051762; Fax: +86-451-51997166.

E-mail: [yangyongbo@caas.cn](mailto:yangyongbo@caas.cn)

Table S1. The nucleotide homology of HLJ13 and represent PRRSV strains

|  | LNWK130 | IA/2014/NADC34 | NADC30 | HEB108 | HUN4 | VR-2332 | QYYZ | ISU30 |
| --- | --- | --- | --- | --- | --- | --- | --- | --- |
| NSP1 | 93.5 | 90.6 | 83.6 | 82.5 | 81.8 | 82.5 | 81.7 | 92.5% |
| NSP2 | 93.7 | 94.2 | 77.2 | 76.7 | 68.3 | 70.2 | 66.2 | 81.0% |
| NSP3 | 96.7 | 96.5 | 85.6 | 86.7 | 81.1 | 83.7 | 79.2 | 86 |
| NSP4 | 94.1 | 95.1 | 79.1 | 81.5 | 85.5 | 83.8 | 80.4 | 90.4 |
| NSP5 | 94.1 | 90.6 | 80.8 | 81.0 | 81.8 | 82.0 | 79.2 | 90.6 |
| NSP6 | 97.9 | 97.9 | 87.5 | 91.7 | 89.6 | 93.8 | 87.5 | 89.6 |
| NSP7 | 94.9 | 94.1 | 80.8 | 82.5 | 79.2 | 81.0 | 78.2 | 88.5 |
| NSP8 | 95.6 | 99.3 | 89.6 | 91.1 | 91.1 | 91.1 | 91.9 | 97 |
| NSP9 | 96.1 | 96.6 | 86.3 | 86.7 | 86.1 | 86.7 | 84.9 | 91.5 |
| NSP10 | 96.0 | 96.4 | 89.2 | 88.7 | 84.7 | 85.7 | 83.9 | 90.9 |
| NSP11 | 97.0 | 97.3 | 86.4 | 85.5 | 86.4 | 85.9 | 86.4 | 91.2 |
| NSP12 | 95.4 | 95.4 | 81.0 | 80.8 | 80.6 | 82.6 | 83.7 | 90.2 |
| ORF2a | 95.7 | 96.8 | 84.2 | 84.8 | 84.7 | 86.9 | 84.4 | 83.8 |
| ORF3 | 94.4 | 97.0 | 82.9 | 83.3 | 82.7 | 83.8 | 82.0 | 84.1 |
| ORF4 | 94.0 | 95.5 | 91.8 | 85.7 | 86.0 | 87.0 | 84.4 | 93.5 |
| ORF5 | 93.9 | 95.0 | 87.2 | 85.9 | 81.6 | 86.6 | 83.6 | 86.1 |
| ORF6 | 94.9 | 95.6 | 90.9 | 90.5 | 87.2 | 87.4 | 87.2 | 91.0 |
| ORF7 | 93.2 | 96.5 | 93.0 | 93.0 | 88.7 | 89.2 | 86.3 | 92.7 |
| Complete genome | 93.3 | 93.7 | 82.2 | 82.1 | 79.0 | 80.0 | 77.8 | 86.3 |

Data were shown as percentage (%)
