# Supplementary material for: Isolation, Pathogenicity, and Comparative Phylogenetic Characteristics of an Intralineage Recombinant NADC34-Like PRRSV in China
Source: Transbound Emerg Dis. 2023 Sep 12;2023:9929573. doi: 10.1155/2023/9929573 (PMC12017108; doi:10.1155/2023/9929573)
Supplement: Supplementary 5 — The homology of amino acid between HLJ13 and represent strains in different lineage and sublineages. [file 9929573.f5.docx]

**Isolation, pathogenicity and comparative phylogenetic characteristics of an intra-lineage recombinant NADC34-like PRRSV in China**

Da-Song Xia^1, #^, Tong Chang^1, #^, Xin-Yi Huang^1^, Xiao-Xiao Tian^1^, Tao Wang^1^, Xing-Yang Cui^1^, Ling-Zhi Luo^1^, Xue-Hui Cai^1,3^, Yong-Bo Yang^1,3, *^, Tong-Qing An^1,2, *^

^1^ State Key Laboratory for Animal Disease Control and Prevention, Harbin Veterinary Research Institute, Chinese Academy of Agricultural Sciences, Harbin, China.

^2^ Heilongjiang Provincial Key Laboratory of Veterinary Immunology, Harbin Veterinary Research Institute, Chinese Academy of Agricultural Sciences, Harbin, China.

^3^ Heilongjiang Veterinary Biopharmaceutical Engineering Technology Research Center, Harbin Veterinary Research Institute, Chinese Academy of Agricultural Sciences, Harbin, China.

* Corresponding author: Dr. Tong-Qing An

State Key Laboratory for Animal Disease Control and Prevention

Harbin Veterinary Research Institute, Chinese Academy of Agricultural Sciences

No. 678 Haping Road, Xiangfang District, Harbin, 150069, China

Tel.: +86-451-51051765; Fax: +86-451-51997166.

E-mail: [antongqing@caas.cn](mailto:antongqing@caas.cn)

Dr. Yong-Bo Yang

State Key Laboratory for Animal Disease Control and Prevention

Harbin Veterinary Research Institute, Chinese Academy of Agricultural Sciences

No. 678 Haping Road, Xiangfang District, Harbin, 150069, China

Tel.: +86-451-51051762; Fax: +86-451-51997166.

E-mail: [yangyongbo@caas.cn](mailto:yangyongbo@caas.cn)

Table S2. The homology of amino acid between HLJ13 and represent strains in different lineage and sublineage

|  | LNWK130 | IA/2014/NADC34 | NADC30 | HEB108 | HUN4 | VR-2332 | QYYZ | ISU30 |
| --- | --- | --- | --- | --- | --- | --- | --- | --- |
| GP2a | 93.8 | 95.7 | 83.0 | 82.5 | 82.5 | 85.2 | 82.5 | 82.9 |
| GP3 | 91.0 | 94.9 | 81.2 | 80.8 | 80.0 | 81.6 | 81.2 | 83.5 |
| GP4 | 92.7 | 96.1 | 93.3 | 87.7 | 89.9 | 88.3 | 86.0 | 93.3 |
| GP5 | 93.0 | 93.5 | 89.1 | 87.6 | 86.6 | 85.6 | 83.6 | 86.6 |
| M | 94.3 | 93.7 | 91.4 | 90.8 | 90.9 | 91.4 | 91.4 | 90.9 |
| N | 90.3 | 95.2 | 92.7 | 92.7 | 88.7 | 87.1 | 86.3 | 91.1 |

Data were shown as percentage (%)
