# Supplementary material for: Isolation, Pathogenicity, and Comparative Phylogenetic Characteristics of an Intralineage Recombinant NADC34-Like PRRSV in China
Source: Transbound Emerg Dis. 2023 Sep 12;2023:9929573. doi: 10.1155/2023/9929573 (PMC12017108; doi:10.1155/2023/9929573)
Supplement: Supplementary 6 — Recombination analysis of NADC34-like strains in China. The preformation was the same as the HLJ13 strain. The left and right sequence of breakpoints were alignment and phylogenetic analysis. The TJnh2021 strain refers to this paper (PMID: 35119777). [file 9929573.f6.docx]

**Isolation, pathogenicity and comparative phylogenetic characteristics of an intra-lineage recombinant NADC34-like PRRSV in China**

Da-Song Xia^1, #^, Tong Chang^1, #^, Xin-Yi Huang^1^, Xiao-Xiao Tian^1^, Tao Wang^1^, Xing-Yang Cui^1^, Ling-Zhi Luo^1^, Xue-Hui Cai^1,3^, Yong-Bo Yang^1,3, *^, Tong-Qing An^1,2, *^

^1^ State Key Laboratory for Animal Disease Control and Prevention, Harbin Veterinary Research Institute, Chinese Academy of Agricultural Sciences, Harbin, China.

^2^ Heilongjiang Provincial Key Laboratory of Veterinary Immunology, Harbin Veterinary Research Institute, Chinese Academy of Agricultural Sciences, Harbin, China.

^3^ Heilongjiang Veterinary Biopharmaceutical Engineering Technology Research Center, Harbin Veterinary Research Institute, Chinese Academy of Agricultural Sciences, Harbin, China.

* Corresponding author: Dr. Tong-Qing An

State Key Laboratory for Animal Disease Control and Prevention

Harbin Veterinary Research Institute, Chinese Academy of Agricultural Sciences

No. 678 Haping Road, Xiangfang District, Harbin, 150069, China

Tel.: +86-451-51051765; Fax: +86-451-51997166.

E-mail: [antongqing@caas.cn](mailto:antongqing@caas.cn)

Dr. Yong-Bo Yang

State Key Laboratory for Animal Disease Control and Prevention

Harbin Veterinary Research Institute, Chinese Academy of Agricultural Sciences

No. 678 Haping Road, Xiangfang District, Harbin, 150069, China

Tel.: +86-451-51051762; Fax: +86-451-51997166.

E-mail: [yangyongbo@caas.cn](mailto:yangyongbo@caas.cn)

**Fig S3**


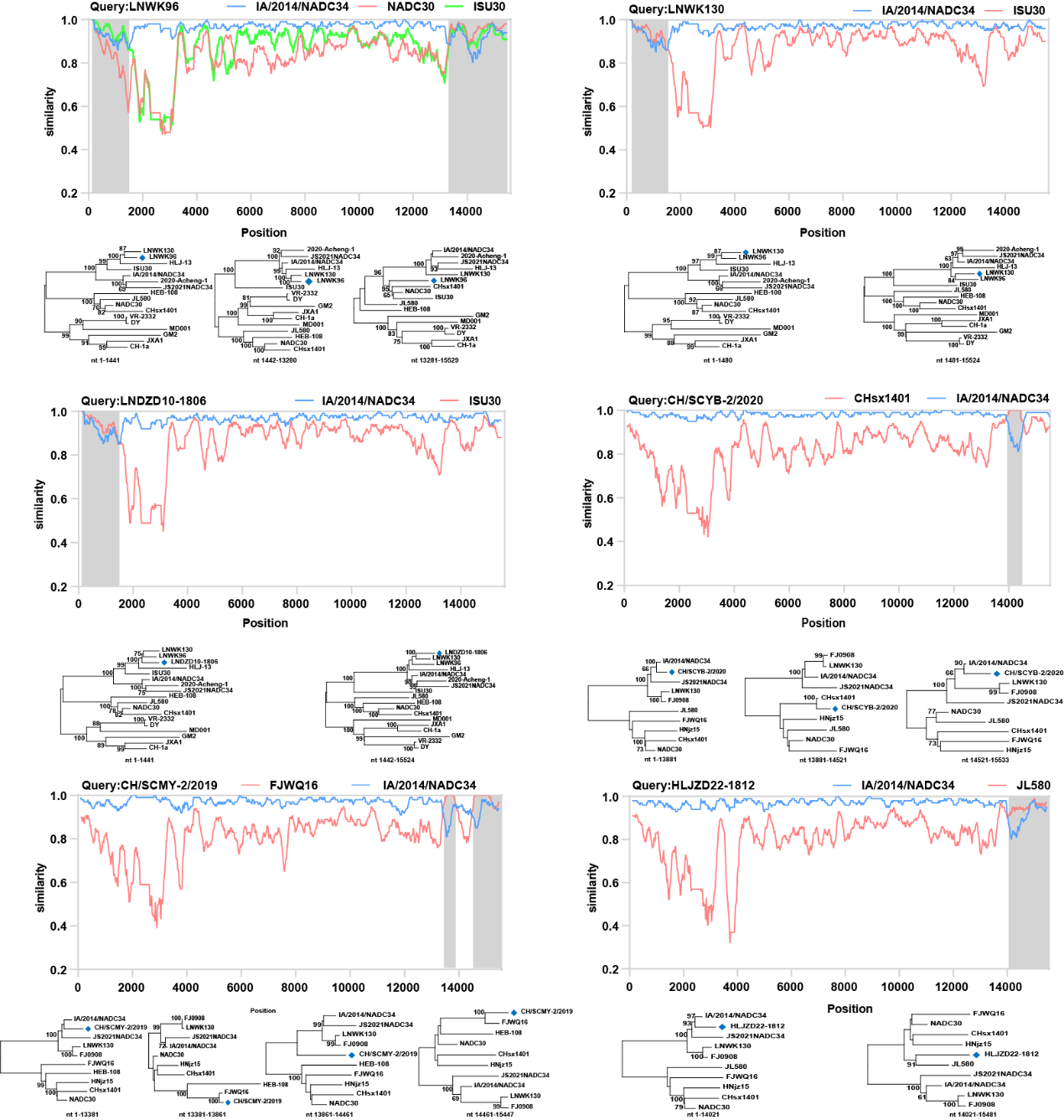


Figure S3: Recombination analysis of NADC34-like strains in China. The preformation was the same as the HLJ13 strain. The left and right sequence of breakpoint were alignment and phylogenetic analysis. The TJnh2021 strain refer to this paper (PMID: 35119777).
